# Histopathological Evidence of Occipital Involvement in Male Androgenetic Alopecia

**DOI:** 10.3389/fmed.2021.790597

**Published:** 2021-11-22

**Authors:** Saranya Khunkhet, Kumutnart Chanprapaph, Suthinee Rutnin, Poonkiat Suchonwanit

**Affiliations:** ^1^Division of Dermatology, Department of Medicine, Faculty of Medicine, Ramathibodi Hospital, Mahidol University, Bangkok, Thailand; ^2^Skin Center, Srinakharinwirot University, Bangkok, Thailand

**Keywords:** pattern hair loss, transverse section, hair count, donor site, hair transplantation, miniaturization

## Abstract

**Background:** The occipital region of the scalp is generally accepted as an unaffected area of androgenetic alopecia (AGA) for both genders. However, evidence of AGA involving the occipital scalp has been demonstrated in women; meanwhile, it is unclear whether occipital involvement also occurs in men.

**Objective:** We aimed to determine if there is occipital involvement in men with AGA.

**Methods:** This case-control study compared hair counts of scalp biopsy specimens from the occipital region of 82 men with Hamilton-Norwood III-VII and 82 unaffected men.

**Results:** The mean ages of men with AGA and controls were 40.1 ± 8.9 and 38.6 ± 10.5 years, respectively (*P* = 0.291). A significant decrease in total hair follicles, terminal hair follicles, follicular units and terminal to vellus (T:V) ratio, along with a significant increase in follicular stelae was indicated in the AGA group compared to controls (all *P* < 0.05). Subgroup analyses revealed that average counts of total hair follicles, terminal hair follicles and T:V ratios were also significantly lower in males with Hamilton-Norwood VI and VII than in controls (all *P* < 0.05). There were no correlations between increasing age and hair count parameters, but a significant negative association was found between total follicle numbers and disease duration (r = −0.23, *P* = 0.02).

**Conclusions:** AGA can involve the occipital area of male patients with advanced disease. Therefore, the occiput of particular cases should not be used to determine reference data for normal scalp hair, and preoperative measurements of miniaturized hairs in the donor site are strongly recommended in all persons undergoing hair transplantation.

## Introduction

The most common cause of hair loss in both men and women is androgenetic alopecia (AGA), also known as pattern hair loss, and is characterized by gradual hair thinning within a specific distribution on the scalp in genetically susceptible individuals (1). Male patients typically present with hairline recession in the frontotemporal areas as well as balding of the vertex and mid-scalp whereas female patients usually manifest as diffuse hair thinning over the central scalp with a preserved frontal hairline (2). Androgens are apparently implicated in the pathogenesis of male AGA; by contrast, the role of androgens in women is much less established (3, 4). Although the etiologies between men and women are not identical, they share similar features which include a miniaturization of hair follicles and a progressive shortening of anagen duration (1, 5).

The diagnosis of AGA is usually straightforward and based upon characteristic clinical findings; however, scalp biopsies, in particular transverse sections, may be required to achieve a definite diagnosis in uncertain cases. The main histopathological features of AGA are as follows: (i) decreased terminal hair follicles and increased vellus hair follicles resulting in a reduced ratio of terminal to vellus (T:V) follicles of <4:1; (ii) a slightly increased telogen count, standardly accounting for 15–20%; (iii) the presence of follicular stelae below miniaturized hair follicles; and (iv) normal follicular density, apart from long-standing and advanced stages, which reveal an actual decrease in total hair follicles (6, 7).

The occipital region of the scalp has been widely accepted as an uninvolved area of AGA for both sexes, under the concept that affected persons have androgen-sensitive and androgen-insensitive scalp regions. This principle has led to the subsequent introduction of donor dominance theory and hair transplantation as a treatment option. Nonetheless, in our practice, we have observed hair thinning extending toward the occipital scalp in some advanced AGA, with more frequent observations among female patients. This may be partly due to the fact that androgen-independent mechanisms also play a role in many women. Moreover, it was highlighted in the field of hair restoration surgery that hair miniaturization can occur in the back and sides of the scalp (the donor sites), which will negatively influence transplant outcomes (8).

Occipital involvement has been demonstrated in female patients with AGA. Previous phototrichogram studies comparing hair characteristics in the occipital area between female AGA and normal controls showed highly consistent results (9–12). Statistically significant decreases in hair density and thickness relative to controls were confirmed in subjects with Ludwig II and III. Additionally, Ekmekci et al. conducted hair counts in biopsy specimens from the mid-scalp and occipital areas of forty female subjects with Ludwig I and II. A quarter of the subjects possessed occipital findings compatible with AGA (T:V ratios of <4:1), and almost 40% of the subjects had findings of suspected AGA in the occipital scalp (T:V ratios ranged from 4:1 to 7:1) (13).

On the contrary, occipital involvement in male AGA has not been proved. The results of phototrichogram studies, performed on the occipital region of men with AGA and normal men, have also been inconsistent. One study showed no significant differences in all hair parameters (14). Another study found statistically significant decreases in hair density and hair diameter in male subjects with the U type of the Basic and Specific classification, corresponding to Hamilton-Norwood VI and VII (15). The aim of our study was to determine whether occipital involvement existed in male patients with AGA.

## Materials and Methods

### Study Design and Participants

This study was approved by the Mahidol University Institutional Review Board for Ethics in Human Research (MURA2020/165) and was conducted in accordance with the principles of the Declaration of Helsinki and in compliance with the International Conference on Harmonization-Good Clinical Practice and local regulatory requirements. Informed consent was obtained from the study participants or their families, as appropriate. All male patients with biopsy-proven AGA, who underwent paired 4 mm punch biopsies from both affected frontal/vertex and clinically normal occipital regions of the scalp for transverse sectioning from 2014 to 2019, were enrolled into this case-control study. Patient demographics and clinical data were collected. Principal exclusion criteria included incomplete medical records and the presence of other hair and scalp disorders. Additional exclusion criteria were documented systemic diseases related to hair loss, the use of medications or energy-based devices which can affect hair growth within 6 months and a history of hair transplantation.

As being considered the most reliable method, histological examination of transverse sections from scalp biopsy specimens was chosen to confirm androgenetic changes. Regarding the biopsy protocol in patients with AGA at our institution, the biopsy landmark on the occipital scalp was the external occipital protuberance of the skull. Paired biopsy specimens from the balding and non-balding occipital scalp were horizontally sectioned using the techniques described by Whiting DA (6). Tissue slides of the biopsy specimen from the occipital scalp in each study subject were reviewed. Two cases were excluded as we were unable to obtain the slides and three cases were excluded on account of tangential cuts, which complicated histologic interpretation. A total of 82 cases remained for analysis.

### Control Subjects

The control group consisted of 82 male adult decedents who required an autopsy for legal documentation at our institution. These decedents had no type of hair loss and received a 4 mm punch biopsy on the normal occipital scalp, following our AGA biopsy protocol, for transverse sectioning within 8 h of death to avoid autolysis, after receiving written informed consent from the bereaved relatives. Controls were randomly selected from a pool of male deceased subjects in our previous studies on hair counts from scalp biopsy specimens in the Thai population (16, 17), with an equal number of cases and controls. The biopsy specimens of controls were processed using a protocol identical to those of the study cases. The exclusion criteria used were also indistinguishable.

### Assessments

Histology slides of specimens from the occipital scalp of both case and control subjects were re-examined by a blinded dermatopathologist. All follicular structures were identified at various anatomical levels from the epidermis to subcutis. The numbers of total, terminal, and vellus hair follicles, along with the number of follicular units, were recorded. Anagen and telogen hair follicles, as well as follicular stelae were also counted. Catagen hair follicles were included in the group of telogen hair follicles. Half of the intermediate hair follicles were assigned to terminal hair follicles with the other half to vellus hair follicles. Differences in hair parameters between groups were subsequently analyzed.

### Statistical Analyses

The minimum sample size for estimation of population mean with a 10% margin of error was 26, calculated using data from the previous study assessing values of hair counts per 4 mm diameter punch biopsy from the occipital scalp of Koreans, demonstrating that the mean number of total hair follicles in male subjects was 15.3 ± 3.9 (18).

Comparisons between study and control groups were performed using the Student's *t*-test, the Mann-Whitney *U* test, or the Chi-squared test as appropriate. For further analyses among study subgroups based on disease severity was conducted using the analysis of variance, the Kruskal-Wallis test, or the Chi-squared test. When the overall comparison *P* < 0.05, pairwise comparisons of subgroups were performed using the Turkey's honest significance difference test, the Mann-Whitney *U* test, or the Chi-squared test as appropriate. The impact of advancing age on hair changes was determined using the Pearson correlation coefficient. A *P* < 0.05 was considered significant, and all statistical analyses were performed with SPSS software (PASW version 18.0; SPSS Inc., Chicago, IL).

## Results

### Comparisons of Hair Count Parameters Between AGA and Control Groups

Hair counts from the clinically normal occipital scalp of patients with AGA, compared to controls, are shown in Table 1. The mean age of 82 patients with AGA was 40.1 ± 8.9 years whereas the mean age of 82 control subjects was 38.6 ± 10.5 years. The age difference between groups was not statistically significant (*P* = 0.291), representing only 18 months; therefore, an age adjustment was not applied. The mean number of total hair follicles in AGA subjects was 17.6 ± 4.2 (95% confidence interval [CI]: 16.68–18.52), showing a significantly lower number compared with controls (mean: 19.1 ± 6.1, 95% CI: 18.56–21.24, *P* = 0.005). Average counts of terminal hair follicles and follicular units were also significantly lower in patients with AGA than in controls (*P* = 0.001 and 0.002, respectively). In addition, there was a significant decrease in T:V ratio and a significant increase in follicular stelae in the AGA group (*P* = 0.001 and 0.035, respectively).

**Table 1:** Hair counts per 4 mm diameter punch biopsy from the occipital scalp of male patients with androgenetic alopecia and controls.

	**Controls**	**AGA**	***P*-value**
Number of cases	82	82	
Age, y, mean (SD)	38.6 (10.5)	40.1 (8.9)	0.291
Total hair follicles, mean (SD)	19.9 (6.1)	17.6 (4.2)	0.005^*^
Terminal hair follicles, mean (SD)	17.9 (4.2)	15.9 (3.8)	0.001^*^
Vellus hair follicles, median (range)	2 (0–7)	3 (0–14)	0.359
Follicular units, mean (SD)	9.3 (1.9)	8.4 (1.8)	0.002^*^
Follicular stelae, median (range)	1 (0–4)	3 (0–12)	0.035^*^
Terminal:vellus ratio	8.9:1	7.4:1	0.001^*^
Anagen:telogen ratio	92.2:7.8	87.6:12.4	0.889

*AGA, androgenetic alopecia; SD, standard deviation; y, year*.

* *Statistically significant*.

### Subgroup Analyses of Subjects With AGA

The summary of hair counts in the control and AGA subgroups based on disease severity are exhibited in Table 2. As for the severity of AGA according to Hamilton-Norwood classification, 14 (17%), 18 (22%), 15 (18%), 19 (23%), and 16 (20%) patients were grades III, IV, V, VI, and VII, respectively. The overall comparisons among subgroups revealed statistically significant differences in total hair follicles, terminal hair follicles, follicular units and T:V ratios (*P* = 0.002, 0.002, 0.035 and 0.001, respectively).

**Table 2:** Hair counts in the occipital scalp of controls and patient subgroups with different disease severity of androgenetic alopecia.

	**Controls**	**Hamilton-Norwood stages of AGA subjects**					***P*-value**
		**III**	**IV**	**V**	**VI**	**VII**	
Number of cases	82	14	18	15	19	16	
Age, y, mean (SD)	38.6 (10.5)	40.5 (11.4)	38.2 (12.4)	44.6 (10.2)	38.8 (8.4)	49.5 (7.4)	0.079
Disease duration, y, mean (SD)	–	3.6 (0.8)	5.2 (1.5)	6.5 (2.2)	6.9 (1.9)	8.3 (2.3)	0.001^*^
Total HFs, mean (SD)	19.9 (6.1)	18.6 (4.9)	19.1 (5.2)	17.8 (5.1)	15.3 (5.4)	14.2 (4.4)	0.002^*^
Terminal HFs, mean (SD)	17.9 (4.2)	17.9 (4.8)	18.3 (4.9)	16.1 (5.2)	13.1 (4.2)	11.9 (3.9)	0.002^*^
Vellus HFs, median (range)	2 (0–7)	2 (0–5)	2 (0–8)	3 (0–10)	3 (0–11)	4 (0–14)	0.146
Follicular units, mean (SD)	9.3 (1.9)	8.7 (1.9)	8.8 (2.1)	8.5 (1.6)	7.9 (1.4)	8.1 (2.5)	0.035^*^
Stelae, median (range)	1 (0–4)	1 (0–4)	1 (0–6)	2 (0–8)	2 (0–7)	3 (0–12)	0.294
Terminal:vellus ratio	8.9:1	8.6:1	8.8:1	7.9:1	6.8:1	5.3:1	0.001^*^
Anagen:telogen ratio	92.2:7.8	90.7:9.3	91.2:8.8	88.6:11.4	85.4:14.6	83.2:16.8	0.294

*AGA, androgenetic alopecia; HF, hair follicle; SD, standard deviation; y, year*.

* *Statistically significant*.

**Figure 1:**
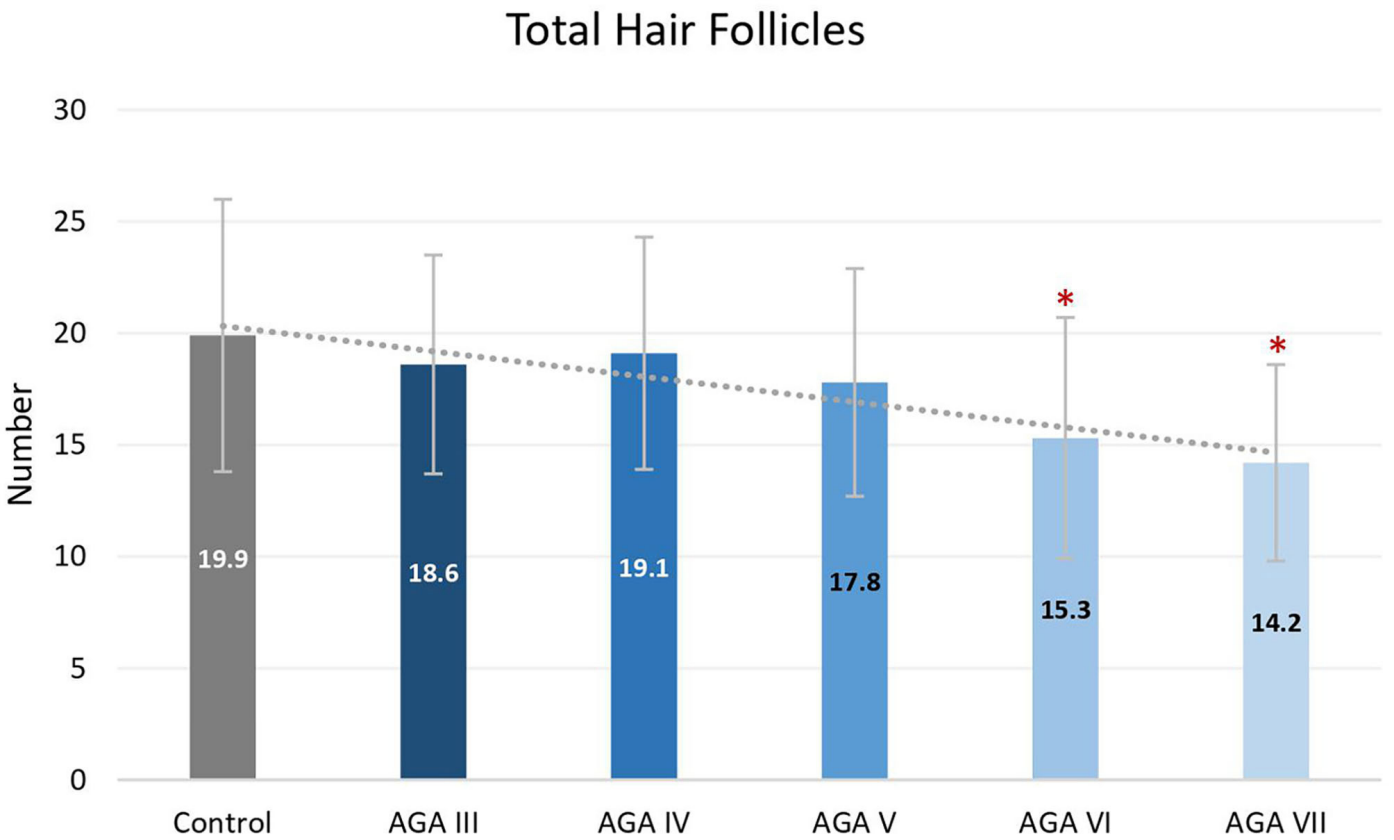
Total hair follicles between controls and patient subgroups with different disease severity of androgenetic alopecia **(AGA)**. *P < 0.05 when compared to the control group.

**Figure 2:**
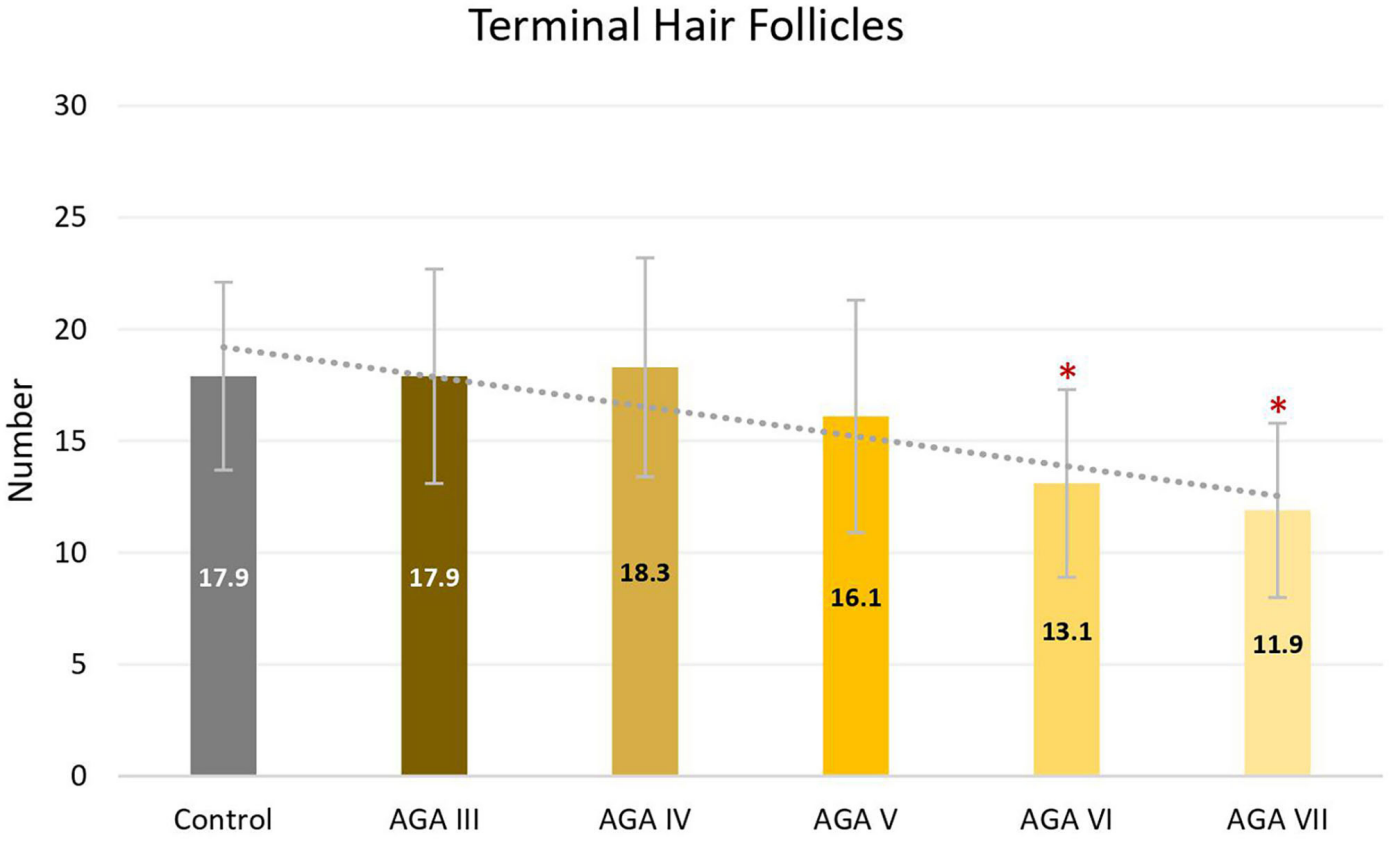
Terminal hair follicles between controls and patient subgroups with different disease severity of androgenetic alopecia **(AGA)**. *P < 0.05 when compared to the control group.

**Figure 3:**
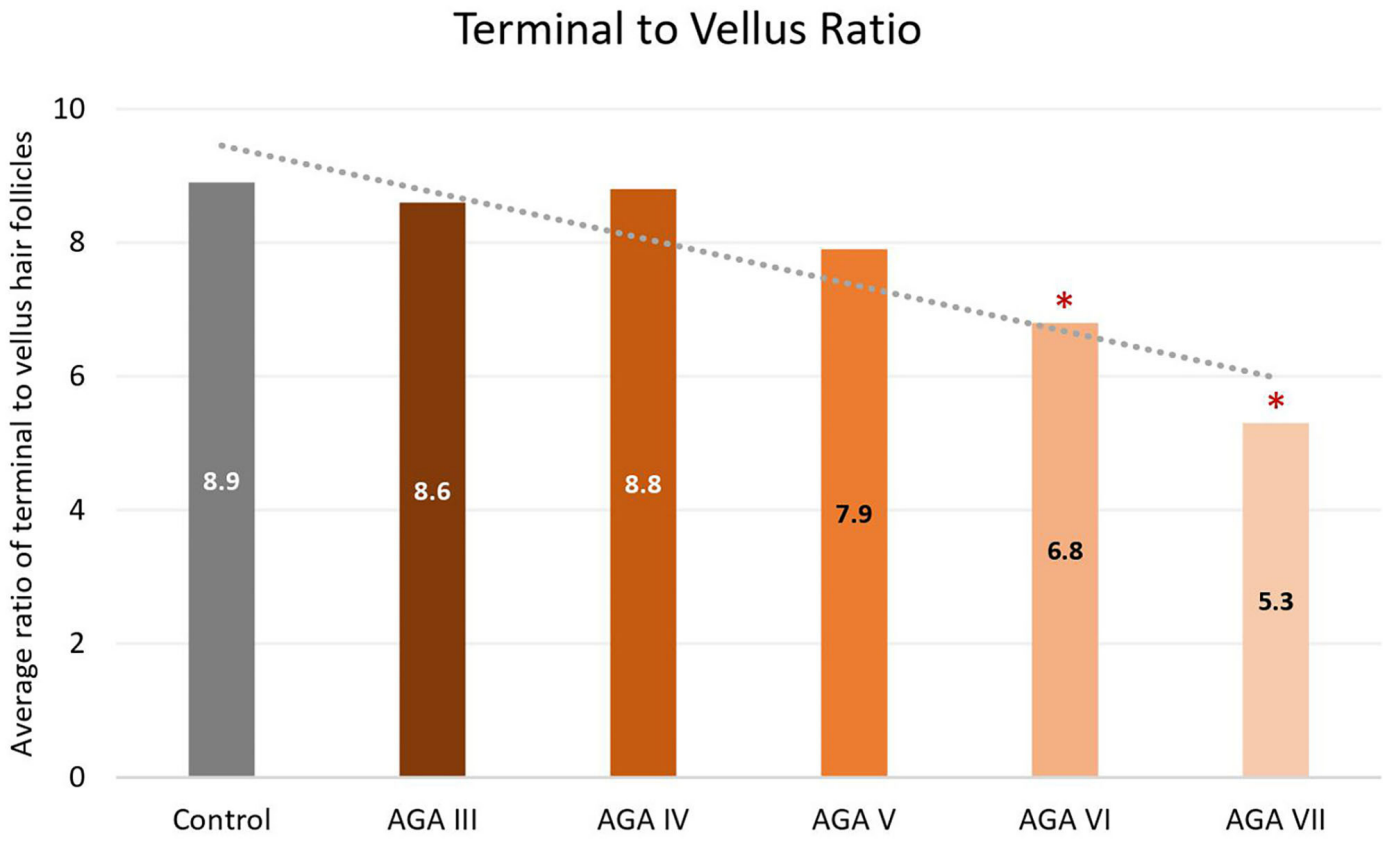
Terminal to vellus hair ratio between controls and patient subgroups with different disease severity of androgenetic alopecia **(AGA)**. *P < 0.05 when compared to the control group.

Subgroup analyses showed no significant differences in age between groups; nevertheless, pairwise comparisons displayed significant decreases in the average numbers of total and terminal hair follicles, as well as T:V ratios, in patients with AGA grades VI and VII, when compared to the control group (all *P* < 0.05) (Figures 1–3). There were no significant associations between increasing age and changes in any of the hair parameters; however, a significant negative association between total follicle numbers and disease duration was found (r = −0.23, *P* = 0.02).

## Discussion

We assessed the histopathological changes in the occipital scalp of men with AGA compared with the normal occipital scalp of unaffected men and found a significant reduction in total hair follicles, terminal hair follicles and T:V ratio, along with a significant increase of follicular stelae in the occipital scalp of male subjects with AGA. Furthermore, AGA patients had a somewhat wider range of vellus follicle numbers than unaffected men, even though a significant difference in statistics was not achieved among median values. These results parallel the findings from previous histopathological studies analyzing biopsy specimens from the frontal or vertex area of men with AGA compared to the same scalp sites of normal controls (6, 19, 20).

Based on subgroup comparisons, the more advanced the AGA, the more pronounced the changes in hair parameters, but statistically significant differences from controls were established only in subgroups with advanced disease (Hamilton-Norwood VI-VII). These findings are consonant with two previous comparative studies using phototrichogram analysis on the occipital scalp between male AGA and normal controls. The study showing no significant differences solely recruited subjects with mild to moderate severity (Hamilton-Norwood I–V) (14), whereas the other study revealed a significant change of hair density and diameter in patients with the U type, the most severe type of the Basic and Specific classification, compatible with Hamilton-Norwood VI–VII (15).

Androgenetic changes in the occipital region appear dissimilar to changes in the frontal and vertex regions. An approximate 25% reduction of total follicle numbers in patients with Hamilton-Norwood VI–VII was noticed while the average T:V ratios in these patients were 6.8:1 and 5.3:1, reduced from a normal ratio of 8.9:1 in controls. A noticeable depletion of total hair follicles can be observed in conjunction with an unsubstantial degree of hair miniaturization. In contrast to changes in the frontal and vertex regions, an actual reduction of total follicle numbers is particularly recognized at advanced stages of disease, when a reversal of terminal to vellus hair follicles seems to be obvious (6, 19). Hence, these feature deviations may imply unique characteristics of the occipital scalp in the process of AGA. We hypothesize that occipital hair follicles comprise two populations: one small component influenced by AGA and the unaffected majority. Therefore, clinical changes of AGA in the occipital scalp can be detected only in very advanced disease when the small population experiences pronounced androgenetic changes, including follicular dropout. Meanwhile, the impact on the T:V ratio remains minimal as a large number of normal hair follicles from the unaffected population are included in the ratio calculation.

Distinct anatomical structures and properties related to androgens are suspected to be mainly responsible for site-specific alterations in the occipital scalp of individuals with AGA (21, 22). Accumulating evidence suggests that hair follicles in the occipital area might elicit less androgen-regulated cellular responses than hair follicles in the other scalp areas (22, 23). Frontal hair follicles contained a significantly higher level of androgen receptors and 5α-reductase enzymes, known for converting testosterone into a more potent androgen called dihydrotestosterone, compared to occipital hair follicles for both genders (24–27). Scalp biopsies also demonstrated a greater amount of aromatase, the enzyme accountable for lowering androgen levels by turning into estrogens, within the occipital rather than the frontal hair follicles in both men and women (21, 24, 28). Moreover, distinctive characteristics of the occipital scalp are perhaps due to its unique embryological origin. In regard to structural development of the head in vertebrates, a large portion is of neural crest origin while some posterior parts of the head, including the occipital region, are derived from the mesoderm (29).

As no correlation was established in this study between increasing age and total follicle numbers, the follicular dropout that occurred among the men with AGA seems not to result from senescent alopecia. These male patients also displayed an increase of miniaturized hairs and telogen counts, which are not features of age-related alopecia (30–34). On the other hand, a negative correlation between total follicle numbers and disease duration was indicated, showing a coherence with histopathological features of AGA in the original sites where follicular dropout can be observed in long-standing disease (7, 35).

The limitation of this study includes the limited ethnic population. Physical properties of hair, such as hair shapes and density, differ between ethnicities. Our findings might not be generalized to other racial and ethnic groups. Further well-designed prospective studies with diverse racial subjects are required to confirm the present findings.

## Conclusion

Three main issues and recommendations are derived from this study. First, AGA extendedly involves the occipital area in male patients with advanced disease. Second, androgenetic changes can occur even in the clinically normal occipital scalp; therefore, the occiput of individuals with AGA, especially in advanced stages, should not be used to determine reference data for normal scalp hair. Third, preoperative measurements of miniaturized hairs present in the donor site are strongly recommended in all persons undergoing hair transplantation due to their impact on the success of surgery.

## Data Availability Statement

The raw data supporting the conclusions of this article will be made available by the authors, without undue reservation.

## Ethics Statement

The studies involving human participants were reviewed and approved by the Mahidol University Institutional Review Board for Ethics in Human Research. Written informed consent to participate in this study was provided by the participants' legal guardian/next of kin.

## Author Contributions

PS and KC: conceptualization and writing—review and editing. PS and SR: methodology and validation. PS, KC, and SK: formal analysis. PS and SK: investigation and data curation. SK and KC: writing—original draft preparation. All authors have read and agreed to the published version of the manuscript.

## Conflict of Interest

The authors declare that the research was conducted in the absence of any commercial or financial relationships that could be construed as a potential conflict of interest.

## Publisher's Note

All claims expressed in this article are solely those of the authors and do not necessarily represent those of their affiliated organizations, or those of the publisher, the editors and the reviewers. Any product that may be evaluated in this article, or claim that may be made by its manufacturer, is not guaranteed or endorsed by the publisher.
